# A Versatile Electronic Tongue Based on Surface Plasmon Resonance Imaging and Cross-Reactive Sensor Arrays—A Mini-Review

**DOI:** 10.3390/s17051046

**Published:** 2017-05-06

**Authors:** Laurie-Amandine Garçon, Maria Genua, Yanjie Hou, Arnaud Buhot, Roberto Calemczuk, Thierry Livache, Martial Billon, Christine Le Narvor, David Bonnaffé, Hugues Lortat-Jacob, Yanxia Hou

**Affiliations:** 1 Institut Nanosciences et Cryogénie, University of Grenoble Alpes, INAC-SyMMES, F-38000 Grenoble, France; laurie-amandine.garcon@hotmail.fr (L.-A.G.); mariagenua@hotmail.com (M.G.); yanjiehou@yahoo.com (Y.H.); arnaud.buhot@cea.fr (A.B.); rcalemczuk@yahoo.com (R.C.); thierry.livache@aryballe.com (T.L.); martial.billon@cea.fr (M.B.); 2 Centre National de la Recherche Scientifique, SyMMES UMR 5819, F-38000 Grenoble, France; 3 Commissariat à l’Energie Atomique et aux Energies Alternatives (CEA), INAC-SyMMES, F-38000 Grenoble, France; 4 Institut Néel, F-38000 Grenoble, France; 5 ICMMO/G2M/LCOM, UMR 8182 (CNRS-UPS), LabEx LERMIT, Université Paris-Sud 11, 91405 Orsay CEDEX, France; christine.le-narvor@u-psud.fr (C.L.N.); david.bonnaffe@u-psud.fr (D.B.); 6 Institut de Biologie Structurale, University of Grenoble Alpes, UMR 5075, 38027 Grenoble, France; hugues.lortat-jacob@ibs.fr; 7 Centre National de la Recherche Scientifique, Institut de Biologie Structurale, UMR 5075, 38027 Grenoble, France; 8 Commissariat à l’Energie Atomique et aux Energies Alternatives (CEA), Institut de Biologie Structurale, UMR 5075, 38027 Grenoble, France

**Keywords:** electronic tongues, cross-reactive sensor array, surface plasmon resonance imaging, pattern recognition, continuous evolution landscape, protein, beverages, milk

## Abstract

Nowadays, there is a strong demand for the development of new analytical devices with novel performances to improve the quality of our daily lives. In this context, multisensor systems such as electronic tongues (eTs) have emerged as promising alternatives. Recently, we have developed a new versatile eT system by coupling surface plasmon resonance imaging (SPRi) with cross-reactive sensor arrays. In order to largely simplify the preparation of sensing materials with a great diversity, an innovative combinatorial approach was proposed by combining and mixing a small number of easily accessible molecules displaying different physicochemical properties. The obtained eT was able to generate 2D continuous evolution profile (CEP) and 3D continuous evolution landscape (CEL), which is also called 3D image, with valuable kinetic information, for the discrimination and classification of samples. Here, diverse applications of such a versatile eT have been summarized. It is not only effective for pure protein analysis, capable of differentiating protein isoforms such as chemokines CXCL12α and CXCL12γ, but can also be generalized for the analysis of complex mixtures, such as milk samples, with promising potential for monitoring the deterioration of milk.

## 1. Introduction

At present, the development of sensors with improved performances and new capabilities is driven by the ever-expanding monitoring needs of a variety of gases and liquid species in diverse domains, including environment monitoring such as air/water quality control, the detection of pollution or leaks of hazardous materials for personal and public safety, food safety and quality control, and non-invasive medical diagnostics. In this context, the electronic noses (eNs) and electronic tongues (eTs) have emerged as promising alternatives. They are engineered to mimic the mammalian olfactory system, consisting of an array of low-selective sensors with cross-sensitivity to different species in complex mixtures and using advanced mathematical procedures for signal processing based on pattern recognition and/or multivariate analysis. Herein, eNs refer to cross-reactive sensor arrays dedicated for the analysis of gas samples and eTs for liquid samples. Unlike gas or liquid chromatography, these devices do not provide information on sample composition in detail, but rather give a characteristic fingerprint through pattern recognition, thus allowing the identification of the sample as a whole.

The last three decades have witnessed great progress in the domain of eNs/eTs thanks to the improvement of sensor technology combined with the artificial intelligence approach. When considering the development of eT systems for analysis of liquid samples, the most common technique is based on electrochemical [1,2], potentiometric [3,4], voltammetric [5], amperometric [6], and impedimetric [7,8,9]) sensors. The potential of these systems for applications in the food and beverages industries as well as the pharmaceutical industry have been reported in several reviews [4,10,11,12,13]. In general, most eT systems developed in Europe are based on electrochemical sensors. For example, the leading company in the eN/eTs domain, Alpha MOS (Toulouse, France), has developed an eT based on the ChemFET (Chemical modified Field Effect Transistor) sensor technology using potentiometric measurements. Remarkably, in the past decade, new eT systems have emerged based on mass sensors (multichannel quartz crystal microbalance (QCM) [14,15] and surface acoustic wave (SAW) [16,17,18]) and optical sensors. In particular, in the US, several groups have worked on cross-reactive sensor arrays using optical sensing approaches and novel sensing materials for the analysis of proteins and complex mixtures. For example, colorimetric sensor arrays were developed by two groups: Suslick’s group, based on a series of functional dyes (solvatochromic dyes, pH indicators, and metalloporphyrins) for analysis of different beverages [19,20,21], and Anslyn’s group, by using an array of 29 boronic acid-containing oligopeptide functionalized resin beads for discriminating proteins [22]. Other groups have focused on fluorescence sensor arrays. Hamilton and co-workers designed and prepared synthetic tetra-phenylporphyrin derivatives as differential receptors for protein sensing [23,24]. More recently, Rotello’s group developed a more sensitive system based on nanoparticle-fluorophore complexes. They carried out the identification of proteins, in serum, at physiologically relevant concentrations [25], which is promising for future application of eTs in the diagnosis of disease states. However, until now, most of the eTs’ application domains remain limited. Thus, there is an identified and current need for the development of new measurement methods and for the search of novel sensing materials to promote a more versatile eT system.

Very recently, our laboratories developed a novel eT by combining surface plasmon resonance imaging (SPRi) with cross-reactive sensor arrays [26]. It is well known that SPRi has been widely applied for “lock-and-key”-based biochips to investigate and quantify biomolecules and interactions [27,28]. To the best of our knowledge, our laboratory was the first to use it for nonspecific and cross-reactive sensor arrays. Indeed, the ability to immobilize many receptors (up to hundreds) on the same surface and to monitor the interactions simultaneously with kinetic information, in a real-time and label-free microarray format, is particularly interesting for eT development. In order to largely simplify the preparation of a diverse variety of sensing materials, an innovative combinatorial approach was proposed. The idea was to use a small number of molecules as building blocks (BBs). BBs were defined as small and easily accessible molecules displaying various physicochemical properties; these BBs are hydrophilic, hydrophobic, negatively charged, positively charged, etc. They can be mixed in varying and controlled proportions, and the different mixtures are arrayed to give combinatorial cross-reactive receptors (CoCRRs) with an exceptional potential for rapid growth in diversity. For example, 11 combinations can be obtained using only two BBs mixed in concentrations varying from 0 to 100% in 10% increments, while 66 can theoretically be accessed by adding a third BB. Moreover, such growth can be generalized to n BBs and i % concentration increments, leading to [(100/i) + n − 1]!/[(n − 1)!(100/i)!] potentially different CoCRRs. The obtained eT is capable of generating 3D images as fingerprints for samples, providing useful kinetic information for discrimination and identification purposes. This mini-review will concentrate on diverse applications we carried out with such a versatile eT [26,29,30,31].

## 2. Electronic Tongue Based on SPRi and Cross-Reactive Sensor Array

**Figure 1:**
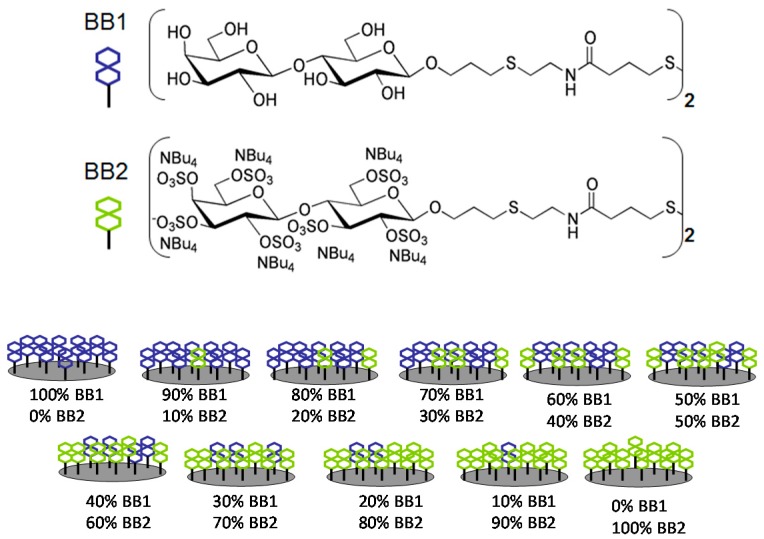
Schematic illustration of the CoCRR array prepared with only 2 building blocks (BBs) such as lactose (BB1) and sulfated lactose (BB2). Reprinted from [30] with permission from JoVE.

For the proof-of-concept study, two small molecules with different physicochemical properties were used as BBs, such as disaccharides lactose (BB1, hydrophilic and neutral) and sulfated lactose (BB2, hydrophilic and negatively charged). A CoCRR array was then constructed, containing 11 cross-reactive receptors made of pure and mixed solutions of BB1 and BB2 at different ratios, as shown in Figure 1.

In practice, at a constant concentration of 20 µM for [BB1 + BB2], 11 pure and mixed solutions with [BB1]/([BB1] + [BB2]) ratios of 0, 10, 20, 30, 40, 50, 60, 70, 80, 90, and 100% were prepared. They were deposited by an automated micro-spotter on a prism surface covered with a thin gold layer. A quadruplicate was deposited for each cross-reactive receptors. The immobilization of the BBs was carried out by the interaction between the disulfide bond and gold surface *via* the formation of self-assembled monolayers (SAMs). Thanks to SAMs, the physicochemical properties of the CoCRRs can be easily modulated by simply varying BB proportions in the mixtures.

**Figure 2:**
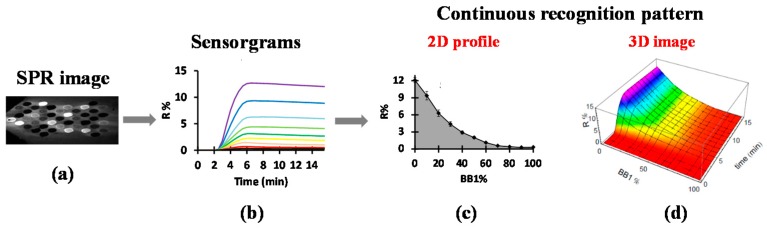
Data treatment for the generation of continuous recognition patterns. (**a**) SPR image recorded by a CCD camera; (**b**) Sensorgrams for all the spots; (**c**) a 2D continuous evolution profile (CEP) and (**d**) a 3D continuous evolution landscape (CEL), also called a 3D image, generated by the electronic tongue (eT). Reprinted from [30] with permission from JoVE.

The SPRi apparatus was placed in an incubator at 25 °C. It was connected to a microfluidic system composed of a syringe pump, a degassing system, a 10 µL PEEK flow cell in a hexagonal configuration, and a 6-port injection valve (for more details, see [30]). The chip containing the CoCRR array was mounted on SPRi device. Upon sample injection on the CoCRR array, molecular binding gave rise to a light-up of spots on the prism with different intensities, as shown in the SPR image (Figure 2a). The signal was then converted to variations of reflectivity (expressed as R%) versus time, yielding sensorgrams composed of kinetic binding curves for all the spots (Figure 2b). Afterwards, based on the sensorgrams, a classical pattern was generated in the form of a histogram by combining the signals obtained with all of the receptors at equilibrium. However, thanks to our combinatorial approach and array design, the composition of each sensing receptor was linked closely to that of its neighbors, giving each receptor a signal that is correlated to that of its neighbor. Thus, all signals generated by the CoCRR array can be considered continuous. In this way, for each sample, we obtained a 2D continuous evolution profile (CEP) by plotting the variation of reflectivity (R%) at equilibrium versus BB1% evolution (Figure 2c). In addition, “time” was added as the third dimension, since SPRi is capable of monitoring the binding events in real time, thereby generating a 3D continuous evolution landscape (CEL), also called a 3D image, as shown in Figure 2d. The 2D CEP and 3D CEL can be used as fingerprints for the discrimination and identification of samples. Moreover, principal component analysis (PCA) was utilized for the classification of samples. Finally, it is important to mention that the obtained eT is reusable after regenerating with an appropriate solution.

## 3. Diverse Applications of the eTs

### 3.1. Analysis of Pure Proteins

**Figure 3:**
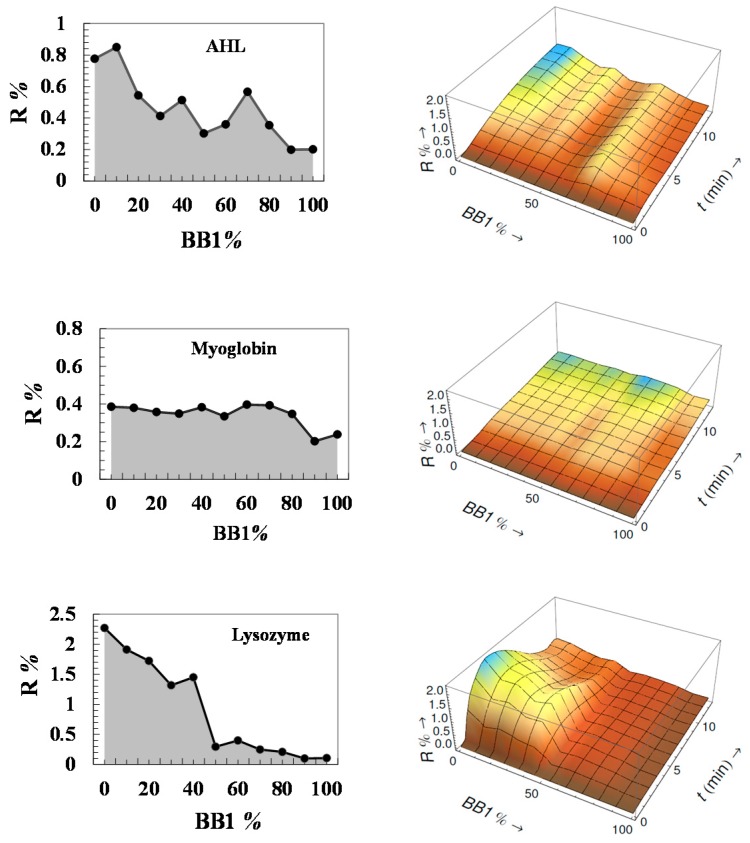
Analysis of common proteins by the eT: CEPs and CELs of *Arachis hypogaea* lectin (AHL) (500 nM), myoglobin (1 µM), and lysozyme (500 nM). Adapted from [30] with permission from JoVE.

Given that the CoCRR array is composed of receptors with different charge densities, we have assumed that such an array can be effective for common protein analysis. For this purpose, a preliminary test was carried out using three proteins, *Arachis hypogaea* lectin (AHL) (isoelectric point (pI) 6.0), myoglobin (pI 7.2), and lysozyme (pI 11). They have different charges under experimental conditions in HEPES (pH 7.4). Satisfyingly, as shown in Figure 3, the eT generated unique response patterns (2D profile and 3D image) for each protein. Based on them, the three proteins were easily distinguishable. For AHL, though slightly negatively charged under such experimental conditions, it has a stronger interaction with the CoCRRs rich in negatively charged BB2, which is possibly due to the interaction between the positively charged domain of the protein and negatively charged CoCRRs. For neutral myoglobin, there is not much difference between its interactions with all of the CoCRRs. As for positively charged lysozyme, as expected, the maximum signal was observed for the CoCRR of pure BB2 with a much higher signal intensity. These preliminary results are very promising, showing that the eT is sufficiently sensitive and selective for the discrimination and the identification of proteins.

It is important to mention that this model array was initially designed by taking inspiration from the way that cell surface heparan sulfates (HSs) recognize HS binding proteins (HSbps). HS are negatively charged polysaccharides with different negatively charged topologies in accordance with cell type and activation state so as to promote selective interactions with HSbps. We hypothesized that the CoCRR array may be more sensitive to HSbps and may promote differential binding for different HSbps, such as chemokines CXCL12α and CXCL12γ. From a structural point of view, they both have the same first part of 68 amino acids, which are folded in a similar manner with a HS binding site (K24-K27-R41) located in a highly structured domain. In addition, CXCL12γ has a second HS binding site composed of 30 amino acids in the unfolded C-terminal extension with good flexibility. For this study, a third protein *Erythrina cristagalli* lectin (ECL) was added. It is a non-HSbp and thus used as a negative control for its binding to CoCRRs rich in BB2. Meanwhile, ECL is known to bind lactose and could thus play the role of a positive reference for the CoCRRs with a high ratio of BB1.

**Figure 4:**
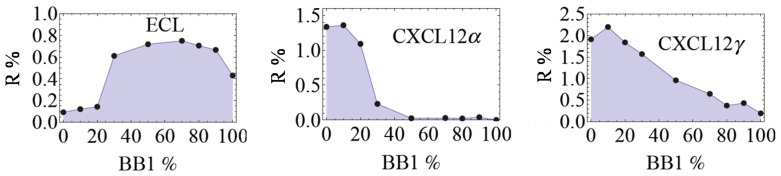
Discrimination of heparan sulfate binding proteins (HSbps) such as CXCL12α and CXCL12γ by the eT. 2D CEPs of ECL (200 nM) used as a non-HSbp for control, CXCL12α and CXCL12γ (both at 100 nM). Adapted from [26].

Upon the injection of these proteins, it was confirmed that the eT was much more sensitive to HSbps and was able to detect CXCL12α and CXCL12γ at low nanomolar concentrations. In Figure 4, the CEPs of the three proteins are given. For ECL, it clearly has a higher affinity for BB1-rich CoCRRs with a maximum for the CoCRR containing 70% of BB1. In contrast, CXCL12α and CXCL12γ have a higher affinity for BB2-rich CoCRRs, displaying maximal signals at 10%. More importantly, the combinatorial surfaces do deliver new and supplementary information compared to the pure ones since the signals of all receptors are non-linear. For instance, it is not possible to differentiate CXCL12α and CXCL12γ based on the signals obtained on the two pure receptors containing 100% of BB1 and 100% of BB2. However, their 2D continuous profiles obtained with all the CoCRRs are clearly distinct from each other. Moreover, for those CoCRRs with the BB1 content at 50% or higher, the obtained reflectivity for CXCL12α is nearly zero. In contrast, CXCL12γ binds significantly on the CoCRRs containing up to 70% of BB1. This can be explained by the difference in global HS binding site(s) topologies and/or rigidity for those two isoforms (see above): the two distant HS binding domains in CXCL12γ can bind in a cooperative way, thus enhancing the affinity for low charge density CoCRRs. Moreover, the second HS binding domains in CXCL12γ is located in an unfolded part of the protein with flexibility. Thus, it could maximize contact points with their ligands through conformational fluctuations.

Thus, from these results, it is evident that the CEPs are correlated with the structures of the proteins so that they are characteristic of the proteins. In consequence, the obtained eT is also efficient for protein identification. Importantly, the continuous evolution of patterns are advantageous when compared with uncorrelated discrete data sets obtained using traditional eT systems, since a defective sensing receptor providing an abnormal signal can be easily identified. Thus, it provides unique “auto-corrective” behavior to the eT.

### 3.2. Analysis of Protein Mixtures

**Figure 5:**
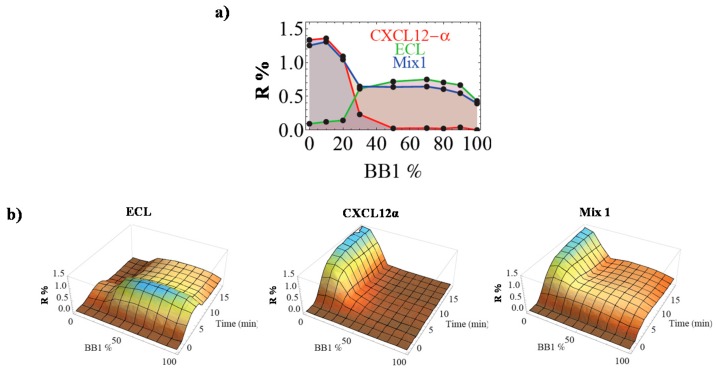
Analysis of simple protein mixtures by the eT. (**a**) CEPs of Mix1 (ECL + CXCL12α) compared to the ones of pure *Erythrina cristagalli* lectin (ECL) and CXCL12α; (**b**) CELs of ECL, CXCL12α, and their mixture Mix1. Adapted from [26].

Furthermore, we have investigated the capacity of the eT for the analysis of protein mixtures. Some preliminary tests were carried out using simple protein mixtures. As an example, CEP of the Mix1 containing 200 nM ECL and 100 nM CXCL12α was shown in Figure 5a together with the CEP of ECL and CXCL12α at the same concentration for comparison. Gratifyingly, the CEP of the Mix1 is distinct from that of the individual pure proteins. Further observations revealed that the CEP of the Mix1 was the combination of the signals obtained from the pure proteins. An identical response was also observed with some other protein mixtures, confirming the potency of the eT for the identification of components in this kind of mixtures by a simple linear decomposition of the CEP into the pure analytes [26].

In addition, as mentioned before, one of the main advantages of SPRi is the ability to monitor the binding events in real time with useful information on adsorption and desorption kinetics, which could serve as a supplementary parameter for sample discrimination. For example, in Figure 5b, the 3D recognition patterns for ECL and CXCL12α are displayed. It is evident that the association phase and dissociation phase of the two proteins on the CoCRR array are different. In particular, the desorption of ECL is much faster than that of CXCL12α. Consequently, discrimination between the two proteins based on the CEL 3D patterns is more straightforward compared to 2D CEP. Thus, such 3D images clearly demonstrate the added value of SPRi for eT development. Furthermore, when compared with the CEL of the Mix1, satisfyingly, it follows the additive behavior found for the CEP. We anticipate that such supplementary discrimination information should facilitate the future identification of analytes in a mixture, since two samples with the same relative affinity for the eT may still differ in their interaction kinetics.

### 3.3. Analysis of Complex Mixtures

Beyond the detection and identification of pure samples, the main goal of the eT technology is to analyze complex mixtures in diverse domains for exploring their potential applications. Quality control of food and beverages is an important issue for both industrial and personal concerns. In the last two decades, eTs have been developed as promising tools in these fields. We thus decided to challenge the capacity of the eT for the study of complex mixtures, such as beverage samples including wines, beers, and milk. Our main concern was whether the 11 sensing receptors, prepared by mixing only two small disaccharides, were able to respond differently to these complex mixtures so as to give good selectivity. Thus, for this study, we attempted to see, on the one hand, whether the eT was able to differentiate between different kinds of beverages and, on the other hand, whether it was able to discriminate different brands of the same species.

**Figure 6:**
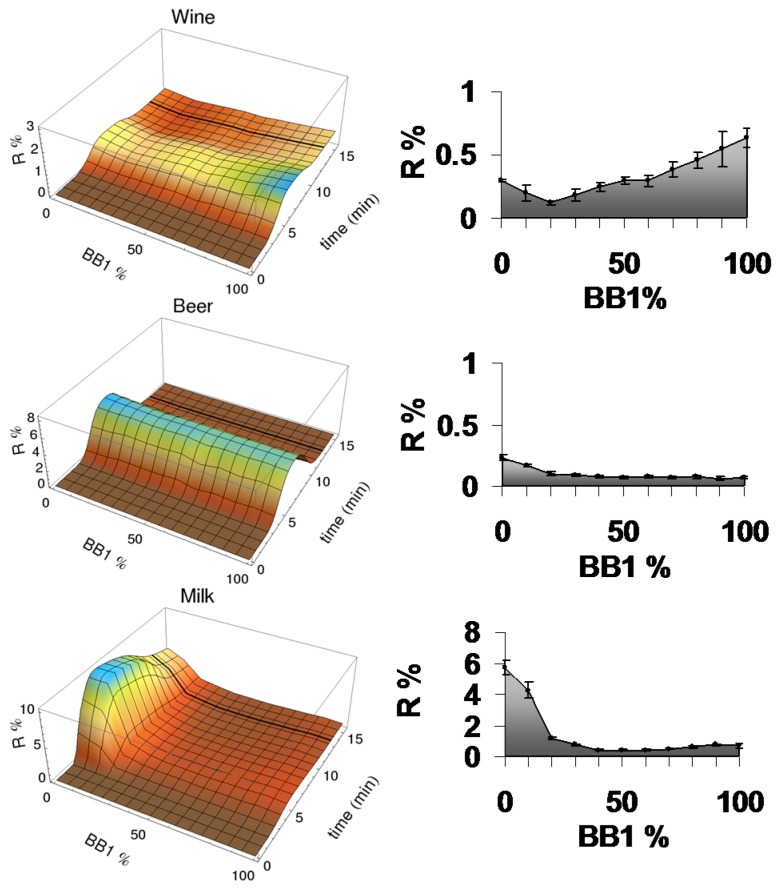
Differentiation of complex mixtures such as beverages by the eT. CEPs and CELs of red wine (Côtes du Rhône), beer (Leffe), and milk (UHT demi-écrémé). Reprinted from [29].

To our delight, the eT responded very differently to these samples with quite good sensitivity, as we can see from their CEPs and CELs, given in Figure 6. The sample of red wine (Bourgogne) had a maximum signal for the CoCRR with 100% BB1. Its CEP was clearly distinct from those of beer and milk. Notably, the milk sample had very low signal intensity on the CoCRRs rich in lactose BB1, which is most likely due to the competition of abundant lactose present in milk. In contrast, its signal intensity on the CoCRRs rich in sulfated lactose BB2 was much higher even using a highly diluted samples, most likely due to the high protein content in the milk sample. These results demonstrated that both their CELs and CEPs can be used as “fingerprints” for differentiation and identification of each sample.

**Figure 7:**
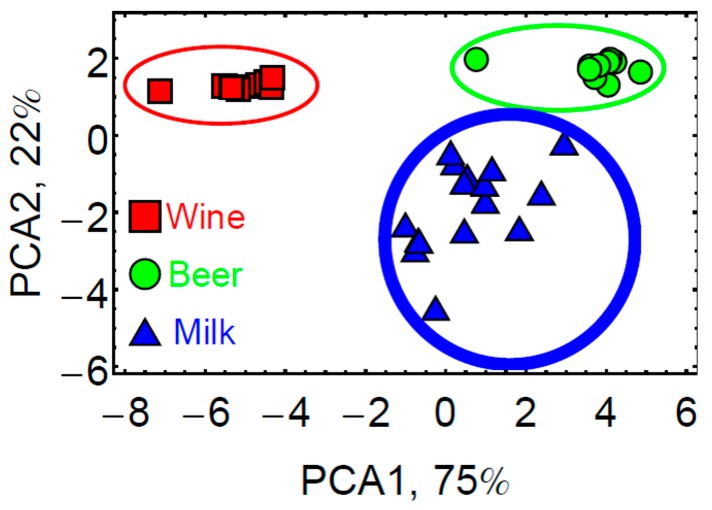
Classification of complex mixtures by the eT based on principal component analysis (PCA) with the two principal components representing 97% of the variance. Reprinted from [29].

Moreover, the eT was used for the classification of a larger number of samples by principal component analysis (PCA). For this study, three different brands of red wines (Côtes du Rhône, Bordeaux and Bourgogne) and beers (Stella Artois, Leffe and Pelforth-dark), as well as UHT milk were analyzed. As shown in Figure 7, the clusters of the three species were well separated using the two principle component axes corresponding to 97% of the variance. However, the eT was not able to differentiate between different brands for wine and beer, which was probably limited by the low signal intensity. This would require the design and introduction of more appropriate BBs.

**Figure 8:**
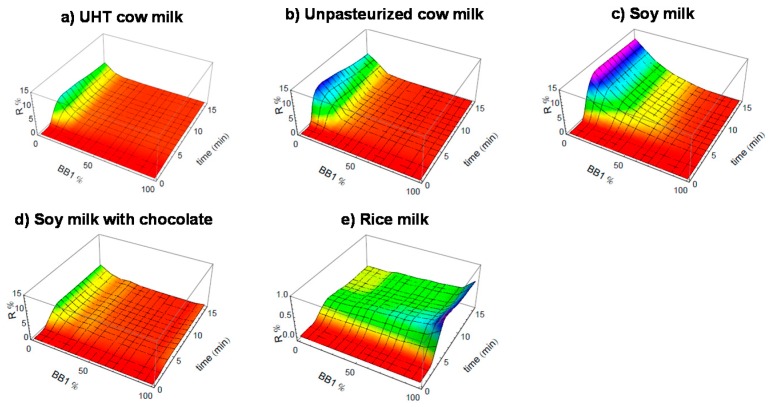
Analysis of protein-rich complex mixtures by the eT. CELs of various milk samples. Adapted from [31].

On the other hand, our eT gave a very good signal for samples rich in proteins like milk. In this regard, the milk sample could be a good model for further study. We then analyzed five different milk samples either animal-based or plant-based, including UHT pasteurized cow milk, unpasteurized cow milk, soy milk, soy milk with chocolate, and rice milk. As demonstrated in Figure 8, their 3D CELs show evident differences. Therefore, these results demonstrated that the eT is very efficient for the analysis of complex mixtures such as milk samples.

**Figure 9:**
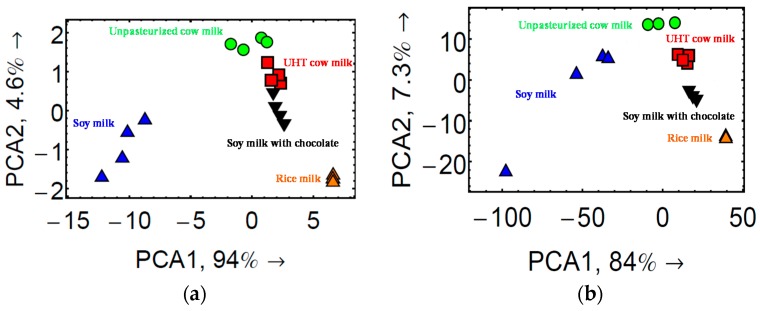
Classification of different milk samples by the eTs with PCA based on 2D CEPs (**a**) and 3D CELs (**b**). For each sample quadruplicate measurements were performed. Reprinted from [31] Copyright@American Scientific Publishers.

To further evaluate the discrimination capacity of the eT, PCA was performed to classify all these milk samples. For this, initially, PCA was performed using the data of 2D CEPs for all 20 milk sample injections with parameters in 11 dimensions corresponding to the 11 CoCRRs. As shown in Figure 9a, there was distinct separation between different milk clusters, except for some overlap between the UHT cow milk and the soy milk with chocolate. However, from their CELs, we can see that the kinetics of cow milk and soy milk are quite different, particularly during the dissociation phase. Thus, we decided to take into account all the kinetic information, including the association/dissociation phase and performed PCA based on 3D CELs. To do so, parameters in more than 300 dimensions were analyzed for each sample using 30 cross sections of CEL, taken every 30 s after the beginning of sample injection for 15 min. Notably, a much better discrimination for all the milk samples was achieved, as shown in Figure 9b. This confirms that 3D CEL-based PCA is more efficient and reliable. Consequently, the kinetic information obtained thanks to SPR imaging is also very important for eT development.

### 3.4. Monitoring Spoilage of Milk

During the experiments for the analysis of milk samples, we observed a quite large distribution of data points in the milk cluster in contrast to those of beers and wines, see Figure 7. This is most likely due to the age difference between the 15 milk samples. In fact, all of them were from the same bottle. So their freshness at the moment they were analyzed was not exactly the same; some were used immediately after opening the bottle, and some others were used after 24 h storage at 4 °C. Thus, it is very probably that the eT is sensitive to the minor changes in such complex mixtures, showing potential for quality control applications. To verify this, we conducted a systematic study to distinguish fresh milk from spoiled milk and to follow the spoilage of milk at room temperature. In practice, immediately after opening, undiluted aliquots of milk samples were stored at 25 °C in an open tube and measured by the eT 1, 24, 48, and 72 h after exposing the sample to air.

**Figure 10:**
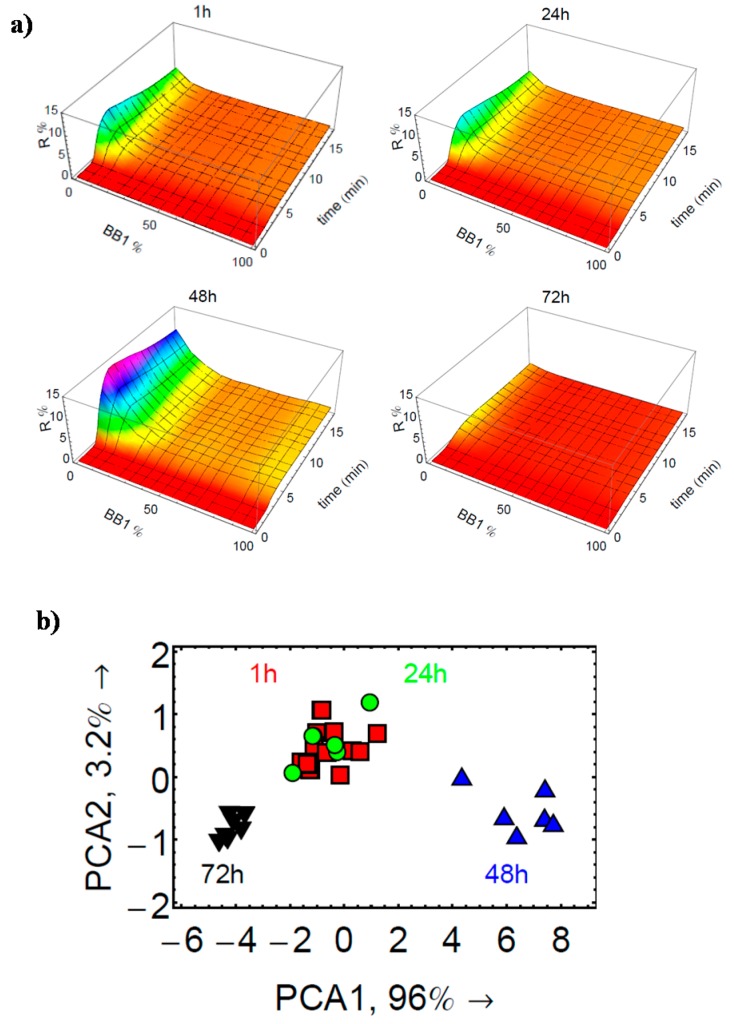
Monitoring spoilage of milk by the eTs: (**a**) 3D CELs of the milk sample in the 1st, 24th, 48th, and 72nd hours after opening; (**b**) PCA score plot derived from the data obtained using these milk samples. Reprinted from [29].

As shown in Figure 10, the CEL of the milk sample 1 h after opening was difficult to distinguish from that obtained after 24 h. There was no major difference on the pattern profile. Nevertheless, it was observed that the signal intensity especially for the CoCRRs rich in BB2 continuously increased over storage time. Therefore, it is most likely that the large distribution of data points in the milk cluster in PCA is due to such signal intensity variation. Satisfyingly, CELs of the 48th and 72nd hours were distinct from those collected in the 1st and 24th hours since there were major modification in the samples in this stage, as confirmed by the PCA plot. According to these preliminary results, the eT is sensitive to the changes associated with the milk spoilage and thus has a potential for quality control applications.

Finally, it is important to mention that the eT demonstrated good repeatability and stability. In practice, for all the applications, pure protein ECL at 200 nM was used as a reference sample and was systematically analyzed at the very beginning, several times in the middle in a random order, and at the end of each analysis set. For each sample to analyze, at least triplicates were used. As reported in our previous work, for both the reference sample and the complex mixture samples, there was a correlation of >99% between any two full patterns of their replicated CEP [29]. A good correlation of >93% was obtained for batch-to-batch reproducibility [26]. Furthermore, the eTs remained very stable under continuous use for at least 50 sample injection/regeneration cycles and for at least two weeks without any loss in sensitivity or sensibility. It had also good stability upon storage at 4 °C over a period of 5 months with no significant loss of signal.

## 4. Conclusions

In summary, a novel eT was developed by combining an innovative combinatorial approach, which simplifies largely the preparation of sensing materials, with the optical detection technique of SPR imaging. Such an eT approach presents certain advantages over existing eT methods. Firstly, based on the combinatorial approach, a great diversity of sensing receptors can be obtained without increasing the cost of the synthesis of new molecules. Secondly, the continuous evolution of patterns (such as 2D profiles and 3D images) are advantageous compared to uncorrelated discrete data sets obtained using traditional eT systems. Abnormal signals obtained with a defective sensing receptor can be easily identified and excluded if necessary, providing a unique “auto-corrective” behavior to our eT system. Third, thanks to SPR imaging, the eT is able to provide a temporal response with vivid 3D images as “fingerprints” for the samples, giving valuable information on adsorption and desorption kinetics. We have demonstrated that the eT is not only effective for pure protein analysis, especially with the ability to differentiate protein isoforms with good sensitivity and selectivity, but can also be generalized for the analysis of complex mixtures with promising potentials for quality control applications. In the future, new building blocks with complementary physico-chemical properties will be designed and introduced to greatly improve the performances of the device.
